# Host Diet Preference Drives Diversity and Composition of Gut Microbiota in Captive Birds

**DOI:** 10.1002/ece3.72463

**Published:** 2025-11-11

**Authors:** Jan Kubovčiak, Jakub Kreisinger

**Affiliations:** ^1^ Department of Zoology, Faculty of Science Charles University Prague Czech Republic; ^2^ Institute of Vertebrate Biology, Czech Academy of Sciences Brno Czech Republic

**Keywords:** 16S rRNA, bird gut microbiota, gut microbiota composition, gut microbiota diversity, host diet and microbiota, host microbiota interactions

## Abstract

Gut microbiota (GM) plays a vital role in host physiology, yet our understanding of the factors driving GM variability in birds remains incomplete. Previous research has provided mixed support for different predictors of bird GM variation, possibly due to the high heterogeneity of avian GM combined with the strong influence of environmental factors on its composition. To suppress the role of these confounding factors, we focused on interspecific GM variation in birds from captive populations, with the aim of clarifying the role of diet and phylogeny. Using 16S rRNA amplicon sequencing, we analysed the GM of 36 bird species from 14 orders, focusing on variability in GM diversity and distribution of individual bacterial constituents. We found that host phylogeny only had limited influence on GM diversity and composition. On the other hand, we identified diet preference of host species as a significant predictor of GM diversity and composition, with herbivorous species exhibiting higher GM alpha diversity than carnivorous species. Furthermore, we observed a converging pattern of GM composition among phylogenetically unrelated carnivorous species, driven by increased abundance of microbial taxa that mostly had an undetermined role in host physiology. This contrasts with obligatory anaerobic bacteria from the phylum *Bacteroidetes*, and other commensal bacteria, observed with increased abundance in hosts preferring carbohydrate‐rich vegetarian diets. Overall, our findings emphasise host diet preference as an important factor determining GM diversity in birds, explaining the convergence of GM composition in phylogenetically distant host species.

## Graphical abstract

We have quantified gut microbiota composition of 36 bird species representing 15 orders to analyse determinants of diversity and variability patterns among host species. We found that host phylogeny only had limited influence on GM diversity and composition. On the other hand, we identified diet preference of host species as a significant predictor of GM diversity and composition, with herbivorous species exhibiting higher GM alpha diversity than carnivorous species.

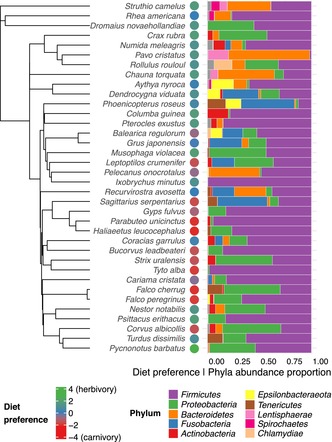

## 1 Introduction

An increasing number of studies emphasise the importance of gut microbiota (GM) in maintaining normal host body functions in vertebrates, with GM having both direct and indirect effects on host physiological systems such as digestion, immunity and maintenance of homeostasis in general (Di Mauro et al. 2013; Hooper et al. 2012; McFall‐Ngai et al. 2013). Most current knowledge on interactions between host and GM is based on studies on mammals; however, research into the composition bird GM has attracted well‐deserved attention in recent years, given the ecological, environmental and economic significance of this group (Cowieson 2022; Deusch et al. 2015; Oakley et al. 2014). Furthermore, the increasing availability and capacity of high‐throughput sequencing technologies in recent years has allowed scientists to elucidate the complex and diverse nature of GM composition in many free‐living bird species under different conditions (Matheen et al. 2022; Sun et al. 2022). This progress has paved the way for investigations into individual factors shaping GM assembly in birds under natural conditions, finally closing the gap between birds and other vertebrate groups such as mammals, which have traditionally received more attention.

Despite numerous studies conducted on birds in recent years, our understanding of the mechanisms that determine variation in their GM is still largely incomplete. It is also becoming increasingly clear that the knowledge we have gained on host–GM interactions in mammals is not readily transferable to birds. Notably, at the interspecific level, host species identity appears to be a stronger determinant of avian GM composition than host ecological traits (Capunitan et al. 2020; Hird et al. 2015). On the other hand, GM in birds shows a relatively weak correlation with host phylogeny compared to mammals (Kropáčková et al. 2017; Perez‐Lamarque et al. 2023; Trevelline et al. 2020; Bodawatta, Koane, et al. 2021). Another crucial mechanism for initial colonisation of offspring by microbiota is the stable transgenerational transmission of microbes from parents to offspring. This process, which mainly occurs during birth and nursing, plays an important role in correlated evolution between microbiota and its mammalian hosts (Coelho et al. 2021; Daft et al. 2015). In oviparous birds, mechanisms that facilitate parental transmission of microbiota are reduced to parental care after hatching and indirect transfer of microbiota from nesting material (Diez‐Méndez et al. 2023), resulting in a lower potential of overall parental effect on offspring microbiota in adulthood (Kreisinger et al. 2017; Maraci et al. 2022); but see Ding et al. (2020).

The effect of diet, the main factor determining GM variation in mammals (Youngblut et al. 2019), has also been investigated in birds, with mixed results (Grond et al. 2019; Loo et al. 2019; Roggenbuck et al. 2014; Schmiedova et al. 2022). Although some studies indicate the importance of specific GM constituents in nutrient absorption (Godoy‐Vitorino et al. 2012) or detoxification (Gunasekaran et al. 2020), these effects are generally weak or even nonsignificant. Nevertheless, the influence of both host species phylogenetic relationships and dietary preferences on GM composition can be overridden by environmental effects, such as geographic location (Hird et al. 2014; Lucas and Heeb 2005), habitat shift due to migration (Lewis et al. 2017; Risely et al. 2018), urbanisation (Murray et al. 2020; Phillips et al. 2018) or seasonal variability (Schmiedova et al. 2023). Such a strong effect of spatiotemporal factors on GM composition makes analysis and interpretation of GM variability in wild birds a challenging task.

Diet and habitat, along with other factors, have been suggested as important contributors to microbiota variability. However, their relative contribution to GM variation in free‐living birds is not yet entirely clear. This might be a consequence of the great diversity in life history traits among birds, whose influence on GM may vary in different clades of avian phylogeny (Sun et al. 2022). Also, designs and methodological approaches in individual studies vary, making it difficult to compare and interpret existing results by means of statistical meta‐analysis. Most studies examining interspecific GM variation in birds have focused on a taxonomically restricted group of hosts, predominantly passerines or other species with a relatively small body size (Matheen et al. 2022). A broader extrapolation of these results to other bird taxa is problematic. Furthermore, the unrepresentative taxonomic coverage of these comparative studies may bias our understanding of factors shaping GM variation across bird phylogeny. For example, convergence of bird GM with that of flying mammals (Song et al. 2020) led to the proposal of a ‘flight‐adapted’ microbiome theory, which assumes that the requirements for flight efficiency represent evolutionary constraints that weaken a host's reliance on beneficial microbial functions provided by fermentative processes (Caviedes‐Vidal et al. 2007). Passerines in particular exhibit such flight‐associated physiological adaptations, e.g., gut reductions (Bodawatta, Koane, et al. 2021), resulting in a looser host–microbiota bond and elevating influence of environmental determinants on GM composition (Schmiedova et al. 2023). Consequently, more representative studies with a broad taxonomic collection of hosts, including both flying and nonflying species, are needed to draw conclusions on the variation and evolution of avian GM. However, this endeavour is fraught with significant caveats related to the nonrandom geographic distribution of avian taxa and the strong dependence of avian GM on environmental factors acting at spatiotemporal scales, which can significantly confound subsequent analyses.

In this study, we aim to overcome the above limitations by systematically selecting a set of species covering most of the taxonomic and ecological range of birds to elucidate how diet and phylogeny influence GM variation. We were able to sample GM profiles from 14 phylogenetically divergent bird orders, showing a range of dietary preferences (e.g., parrots vs. vultures). To attenuate GM variation attributable to environmental factors, we used captive birds from five zoological gardens. Captivity has effect on GM diversity and composition in different mammalian species (McKenzie et al. 2017). In birds, studies showed captivity to be associated with GM diversity decrease in house sparrows ( *Passer domesticus* ) (Florkowski et al. 2023) and shifts in GM composition in kestrels ( *Falco tinnunculus* ) (Zhang et al. 2022) and bar‐headed goose ( *Anser indicus* ) (Wang et al. 2017). However, we argue that, in our case, the benefits associated with the relative homogeneity of environmental factors outweigh the potential limitations associated with the captive environment, granting more robust analyses regarding the effects of diet and phylogeny on GM variability. These, along with the influence of environment, are considered the most important factors shaping GM diversity in birds, though their effect is still controversial according to current empirical evidence.

## 2 Material and Methods

### 2.1 Sampling and Microbiota Genotyping

Sample collection took place at five selected zoological gardens in the Czech Republic (Dvůr Králové nad Labem, Plzeň, Brno, Jihlava, Ostrava) from the beginning of May to the end of October 2019 in the morning. We opted for a noninvasive approach for collecting faecal samples, previously established as a good proxy for actual GM (Schmiedová et al. 2024; Videvall et al. 2018; Yan et al. 2019). Faecal pellets (one sample per individual) were harvested with minimal possible delay after defecation, directly from the aviaries, using sterile microbiological swabs (minitip FLOQSwabs, Copan, Italy) and placed in sterile tubes filled with 99% ethanol. Note that GM composition is not expected to change significantly during short‐term exposure to the environment (Čížková et al. 2021). Nevertheless, negative control samples of surface material (soil, sand or sawdust) were regularly collected (*n* = 40), applying the same routine used for faecal pellets. Subsequently, these samples were used to identify and filter potential environmental contaminants from the resulting GM profiles. Within 2 days of collection, the samples were transferred to a freezer and stored at −20°C until further processing. In total, faecal samples were collected from 119 adult individuals representing 40 bird species from 15 orders. Composition of rations fed to individual species in respective zoological gardens is provided in Table S1B.

Metagenomic DNA from faecal and negative control samples, together with blank DNA isolates (*n* = 5), were extracted using the DNeasy PowerSoil DNA isolation kit (Qiagen, Netherlands). Sequencing libraries were prepared using a two‐step PCR protocol, as described in Čížková et al. (2021). The first PCR used the standard metabarcoding primers of Klindworth et al. (2013) targeting the V3–V4 region of the bacterial 16S rRNA gene. For the second PCR, the primers were extended by inline barcodes and priming sites, where dual indexes and Illumina‐compatible, Nextera‐like sequencing adapters were added. Each metabarcoding PCR was performed in technical duplicates to account for PCR and sequencing stochasticity. Positive PCR control samples (*n* = 2) containing DNA isolates of a bacterial mock community (ZymoBIOMICS Microbial Community DNA Standard) were then introduced. The PCR products were pooled based on their concentration and sequenced using the Illumina MiSeq platform with v3 kit with 2 × 300 bp paired‐end reads configuration at the Central European Institute of Technology (CEITEC; Brno, Czech Republic).

**FIGURE 1:**
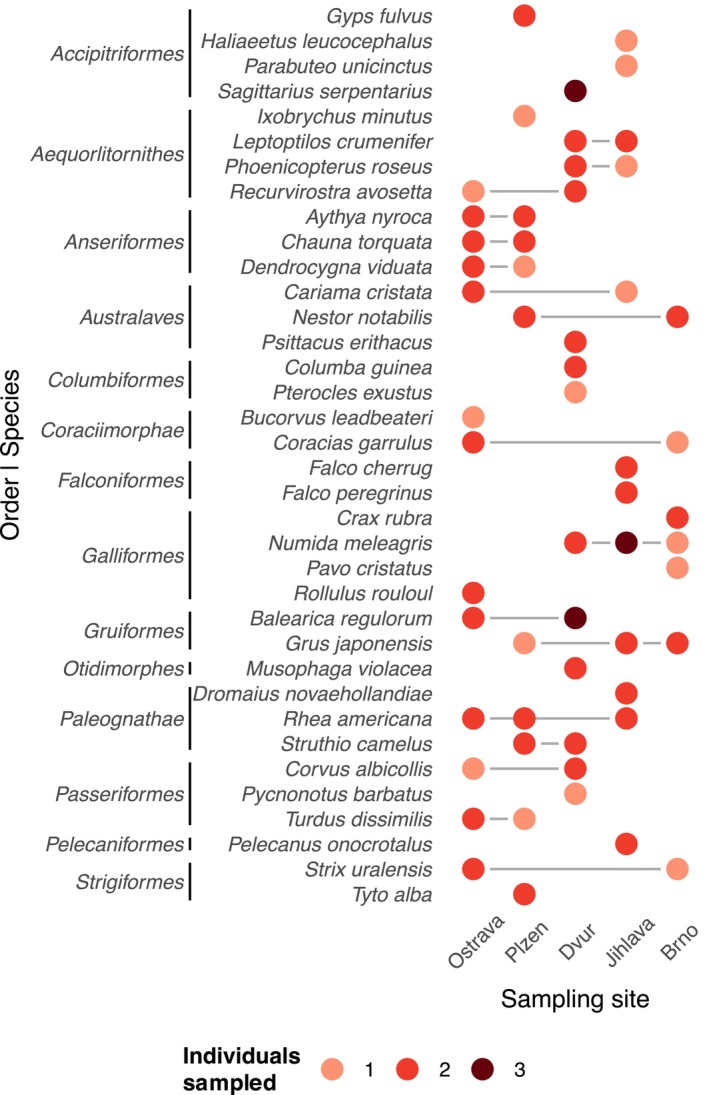
Counts of individuals analysed by species and sampling site. Host species and orders sorted alphabetically.

The resulting fastq files were demultiplexed based on sample‐specific barcodes, and 16S rRNA primers detected and trimmed using Skewer (Jiang et al. 2014). The R package DADA2 (Callahan et al. 2016) was used to eliminate low‐quality reads (expected error rate per paired‐end read > 2), to denoise the quality‐filtered reads, and to generate abundance matrices of read counts for each 16S rRNA amplicon sequencing variant (ASV) in each sample. Subsequently, the UCHIME algorithm (Edgar et al. 2011) was used to detect and eliminate chimeric ASVs using the ‘Gold’ database (available at: https://drive5.com/uchime/gold.fa) as a reference. Taxonomy assignment for non‐chimeric ASVs was performed using the RDP classifier (Wang et al. 2007), with 80% posterior confidence and the Silva database v.138 (Quast et al. 2013) used for bacterial ASV annotations. Data were then converted to phyloseq‐class format (McMurdie and Holmes 2013) for subsequent analyses in the R program (R Core Team 2015). In the next step, ASVs annotated as Archaea, mitochondria or chloroplasts were discarded, as were ASVs unassigned at the kingdom or phylum level. ASV profile consistency between technical duplicates was assessed using Procrustes analysis (Legendre et al. 2011). The duplicated data were then merged, after first eliminating ASVs undetected in both duplicates to further reduce PCR and sequencing artefacts. The R package decontam (Davis et al. 2018) was then used to identify and filter contaminating ASVs, based on their prevalence in the negative control samples and concentrations of PCR products. We also discarded 21 GM samples with low sequencing depth after all filtering steps (*n* < 500). The final filtered and decontaminated data set contained 98 GM profiles (82% of initial sample count) representing 36 species and 14 bird orders (Figure 1, Table S1A) with total abundance of 525,165 reads and average sample abundance of 5359 reads and 3447 unique ASVs. To normalise the data for different sequencing depths between samples in statistical modelling, sample abundances were rarefied to a constant value of 1100 without replacement, after which 2933 ASVs (85%) were retained. The rarefaction procedure had no effect on exploratory analysis interpretation, and the values of resulting per‐sample alpha diversity indices before and after rarefaction were strongly correlated (Pearson's *r* > 0.9, *p* < 1 × 10^−10^ for both Shannon index and ASV richness).

### 2.2 Statistical Analysis

Similarity of resulting GM profiles was assessed using Principal component analysis (PCoA) computed on a binary version of Jaccard distances between samples. Owing to the low proportion of total variance explained by the first two components (5.7%), uniform manifold approximation and projection (UMAP) embedding (Konopka 2023; McInnes et al. 2020) was generated based on the top 20 PCoA components (explaining 33.8% of total GM variance). UMAP is a nonlinear dimension reduction method, suitable for visualisation of high dimensional data in low‐dimensional space. It models the topology of a high dimensional manifold of the data and calculates embeddings of the individual points that best correspond to relationships between the data on the manifold, retaining more information compared to using just two PCoA components.

We employed phylogeny‐informed mixed‐effect models (Li et al. 2020) to analyse the variance in alpha diversity indices (log_10_ transformed) and joint species distribution modelling approach (Ovaskainen et al. 2017) to analyse the variance in distribution of individual ASVs (beta diversity). For alpha diversity analysis, we also focused on differences among three major host clades (*Neoaves*, *Paleognathae* and *Galloanserae*). The host clade and preferred diet were assumed to be fixed effect factors, while sampling site, host phylogeny and species identity were used as random terms. We also tested a model including sampling site as a fixed explanatory variable to evaluate effect of individual zoological gardens. To accommodate host species evolutionary history, we constructed a consensual phylogenetic tree, calculated using consensus. edges function in the R package phytools (Revell 2012), based on a set of 1000 Bayesian trees with Hackett backbone (obtained from http://birdtree.org/; Jetz et al. 2012). The data on actual diet rations (Table S1B) are difficult to objectively convert into factors suitable for statistical analyses. Thus, as a proxy for host species diet, we used scores for the first principal component of the PCA, computed based on the proportions of dietary components extracted from the Elton Traits Database (Wilman et al. 2014). The first component (explaining 20.6% of the variance) distributed the species along the vegetarian–carnivore scale. It is noteworthy that this approach provides results that largely correspond to the recorded feed rations.

**FIGURE 2:**
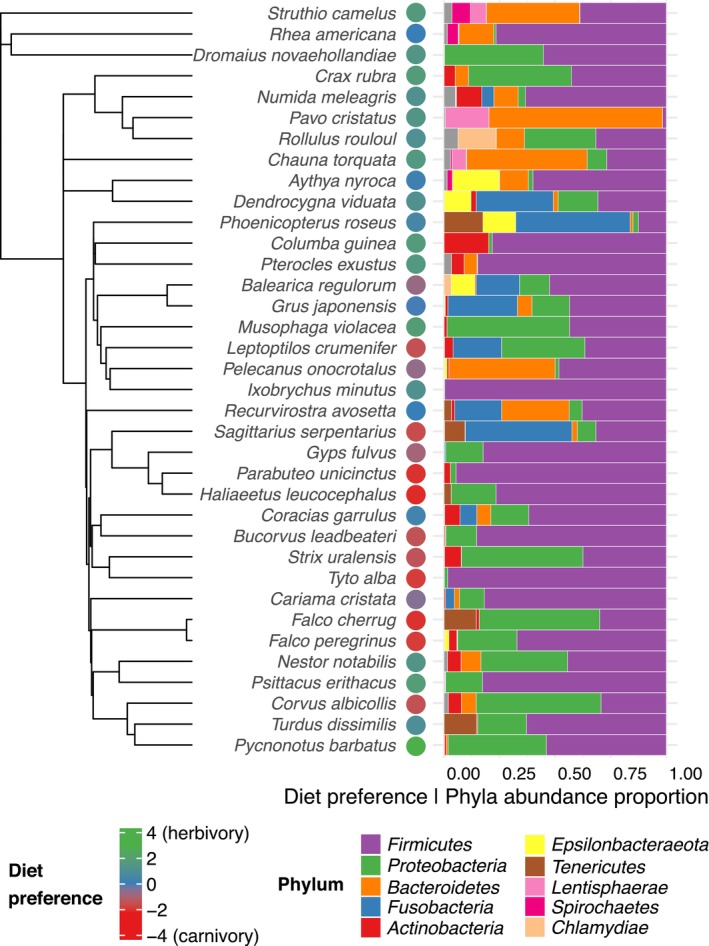
Proportions of bacterial phyla per host species, with a dendrogram representing host species phylogeny and points indicating diet preference.

For beta diversity analysis, we first used PGLMM models to test whether diet or host clade explain variance in abundance of the three most common bacterial phyla (*Firmicutes*, *Proteobacteria* and *Bacteroidetes*, Figure 2). In order to account for compositional nature of metabarcoding based microbial data (Gloor et al. 2017), relative abundances were transformed using robust centred log ratio method prior PGLMM fitting (Martino et al. 2019). Furthermore, we employed ABDOMEN approach (Perez‐Lamarque et al. 2023) to model GM evolution and to test effect of phylosymbiosis between host species phylogeny and GM composition, averaged among individuals from the same host species. Due to the limited number of hosts, sparsity of microbial data, and in line with general recommendations of ABDOMEN developers, we restricted these analyses to the five most abundant GM phyla, still representing 95% of total abundance. We assumed empirical prior on the ancestral abundances (Z0). A Markov chain Monte Carlo (MCMC) parameter estimation procedure was run in two chains with total number of 5000 samples per chain including burn‐in of 1000 samples. We extracted Pagel's *λ*, indicating strength of phylosymbiosis, and covariances between analysed GM phyla. Significance of Pagel's *λ* was assessed using 100 permuted data sets created by randomly shuffling tip labels of host species phylogenetic tree. We also calculated Pagel's *λ* for host–species‐specific diet preference (represented as PC1 scores) to compare with phylogenetic signal of GM composition variability.

To investigate factors determining distribution of individual ASVs, a joint species distribution model of ASVs abundance among samples was constructed using HMSC package (Tikhonov et al. 2022). Only those ASVs present in at least 3% of samples were used to analyse only abundant ASVs represented in GM profiles of multiple individuals. After filtering, the ASVs count was reduced to 147, representing nine of the original 22 phyla while retaining 10.1% of original dataset abundance. We declare, that the reduced data set still represents most of the original variability in beta‐diversity, as exploratory analysis based on this ASV subset yielded similar results compared to the full dataset. Furthermore, Jaccard's intersample distances between full and the reduced dataset still exhibited significant correlation (Pearson's *r* = 0.86, *p* < 2.2 × 10^−16^). We generated two models with (a) per‐sample ASV abundance, assuming log‐normal Poisson distribution, and (b) an abundance matrix transformed to represent ASV presence or absence, assuming probit distribution as a response. We supplied cophenetic distances among samples calculated from the phylogenetic tree described above to quantify effect of host species phylogeny. Preferred diet was taken as an explanatory fixed effect with sampling site, host species identity, phylogeny and individual sample identity as random effects. MCMC parameter estimation procedure was run in two chains with a burn‐in of 900,000 samples, with every 1500th step sampled until the total of 1000 samples per chain was reached. Convergence of the alpha (host phylogeny) and beta (ASV niches) parameters estimation was controlled for autocorrelation using effective sample size estimation, and for potential scale reduction using Gelman diagnostics. As the log‐normal Poisson model failed to converge, probably due to pronounced sparsity of the data, the probit model results only were evaluated further. Interpretation of the model results was based on those parameters whose estimates received considerable support (i.e., deviated from zero with a posterior probability > 0.95). The proportion of variance in the prevalence of each ASV explained by individual variables was calculated in order to compare overall effects of the factors studied.

## 3 Results

### 3.1 Gut Microbiota Composition

**FIGURE 3:**
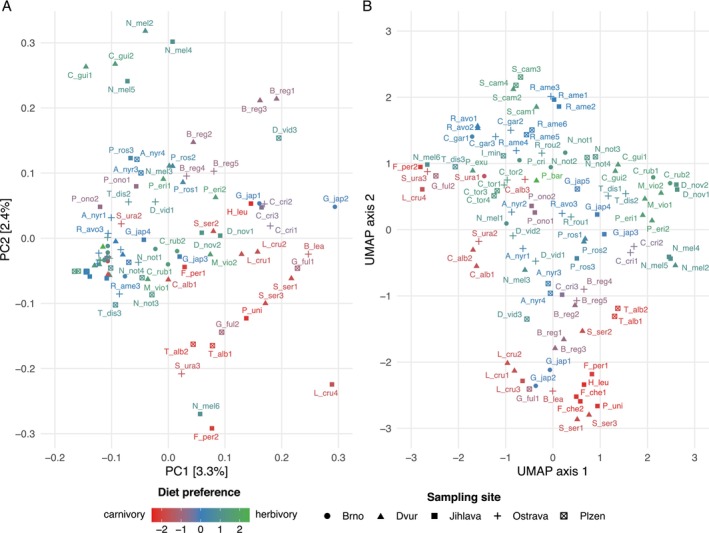
Similarity of gut microbiota (GM) profiles as (A) binary Jaccard distances scaled using a principal component analysis (PCoA) showing the first two principal components, and (B) uniform manifold approximation and projection (UMAP) embedding based on the first 20 principal components explaining the highest proportion of total variance. The colour scale represents host‐species diet preference, ranging from carnivorous (red) to vegetarian (green). See Table S1 for key to the sample labels.

We identified 3447 unique ASVs (mean of 46 ASVs per sample and 152 reads per ASV) from 263 known bacterial genera and 22 phyla. Of these, 60%, representing 83% of total abundance, were successfully assigned to genus level. The most represented phyla were *Firmicutes* (average abundance: 49% ± 31% SE), *Proteobacteria* (18% ± 22%), *Bacteroidetes* (12% ± 22%) and *Fusobacteria* (9% ± 18%) (Figure 2). GM profiles exhibited pronounced taxonomic variability, with just 11 ASVs (0.32% of all ASVs detected) and 25 genera (9.5% of all genera detected) shared between at least 10 individuals. Exploratory analysis using PCoA and UMAP projections (Figure 3) showed noticeable grouping of GM profiles from species sharing a carnivorous diet preference (*Accipitriformes*, *Falconiformes*, marabou stork [*Leptoptilos crumenifer*] and southern ground hornbill [ *Bucorvus leadbeateri* ]), with omnivorous cranes close to this group. On the other hand, some predominantly carnivorous species, for example, the Ural owl ( *Strix uralensis* ) or white‐necked raven ( *Corvus albicollis* ), were clustered together, but separately from other carnivores. No pattern corresponding to sampling site or other technical parameters (collection time, date of isolation) was observed based on UMAP or PCoA (Figure S2A–C).

### 3.2 Alpha Diversity Analysis

Alpha diversity exhibited pronounced differences between sampling sites for both ASV richness and Shannon index (Figure S1, Table S2C); however, this was due to nonrandom sampling of host species with similar diet preferences. Overall, we observed significant increase of Shannon diversity index with vegetarian diet propensity, in contrast to lower GM diversity in carnivorous species (*p* = 0.046; Table S2A). ASV richness exhibited similar, but not significant trend (*p* = 0.065). Sampling site explained most variance in the model based on ASV richness (14% for richness, 9% for Shannon) and host species identity explained most variance in the model based on Shannon index (< 1% for richness, 13.6% for Shannon). Host species phylogeny explained 8.7% for model on ASV richness, and only limited proportion of total variance for model on Shannon index (4.3%). Elimination of phylogenetic correlation from the model resulted in a slightly better model fit (ΔAIC^Shannon^ = 1.94, ΔAIC^richness^ = 0.77), on the other hand, these reduced models exhibited and increased significance for the effect of diet preference (richness *p* = 0.024, Shannon *p* = 0.033; Table 1A).

**TABLE 1:** Results of selected reduced Phylogenetic Generalised Linear Mixed Models explaining variation in alpha diversity indices.

(A) Explanatory: host diet preference					
		Observed (log_10_)		Shannon	
		Variance	SD	Variance	SD
Random effects	Species	0.006	0.075	0.106	0.325
	Sampling site	0.009	0.094	0.053	0.230
	Residual	0.069	0.264	0.412	0.642

		Estimate	SE	*Z*‐score	*p*	Estimate	SE	*Z*‐score	*p*
Fixed effects	(Intercept)	1.527	0.053	29.071	0.000	2.472	0.136	18.125	0.000
	Diet preference	0.055	0.024	2.251	0.024	0.147	0.069	2.134	0.033
AIC		36.46				216.13			

(B) Explanatory: host clade					
		Observed (log_10_)		Shannon	
		Variance	SD	Variance	SD
Random effects	Species	0.001	0.038	0.112	0.334
	Sampling site	0.018	0.134	0.105	0.324
	Residual	0.069	0.263	0.397	0.630

		Estimate	SE	*Z*‐score	*p*	Estimate	SE	*Z*‐score	*p*
Fixed effects	(Intercept)	1.793	0.107	16.765	0.000	3.102	0.320	9.682	0.000
	*Galloanserae*	−0.276	0.107	−2.570	0.010	−0.615	0.348	−1.766	0.077
	*Neoaves*	−0.290	0.094	−3.070	0.002	−0.706	0.303	−2.323	0.020
AIC		35.27				217.37			

*Note:* Values are based on restricted maximum likelihood fit with the exception of AIC for comparison. Significant *p*‐values (< 0.05) are highlighted with a yellow background.

Models including host species clade as an explanatory variable revealed significantly lower (*p* < 0.04) ASV richness in the *Galloanserae* and *Neoaves* groups than in the order *Paleognathae* (Table S2A). Shannon diversity exhibited the same trend, but without statistical significance (*p* = 0.08). Again, reduced models not accounting for phylogenetic correlation showed comparable performance (ΔAIC^Shannon^ = 2, ΔAIC^richness^ = 1.71; Table 1B), while models that included both preferred diet and host clade as explanatory variables showed only a slight improvement in performance compared with models that included just one of these variables (ΔAIC^Shannon^ = 0.49, ΔAIC^richness^ < 2.18). While both Shannon diversity and ASV richness showed no significant response to diet preference in these complex models (*p* > 0.12), host clade continued to exhibit a significant effect (*p* < 0.03) on ASV richness (Table S2A).

### 3.3 Beta Diversity Analysis

PGLMMs for abundance of the three most common GM phyla showed significantly lower abundance of *Bacteroidetes* (*p* = 0.02) in the *Neoaves* clade (Table S2B). In contrast, *Proteobacteria* tended to be nonsignificantly less abundant in the *Paleognathae* clade compared to *Galloanserae* and *Neoaves*. Abundance of *Firmicutes* exhibited no response pattern to preferred diet or host species clade. ABDOMEN analysis for top five most abundant phyla revealed moderate effect of phylosymbiosis (Pagel's *λ*: 0.4, 95% CI: 0.13–0.69, *p* value 0.08). However, estimated GM phyla covariances showed strong negative covariance (95% CI not including 0) between abundance of phyla *Proteobacteria* and *Bacteroidetes*, supporting results of PGLMMs (Figure S4). Prediction of ancestral GM composition shows also reduced proportions of *Bacteroidetes* among *Neoaves* (Figure S3).

**FIGURE 4:**
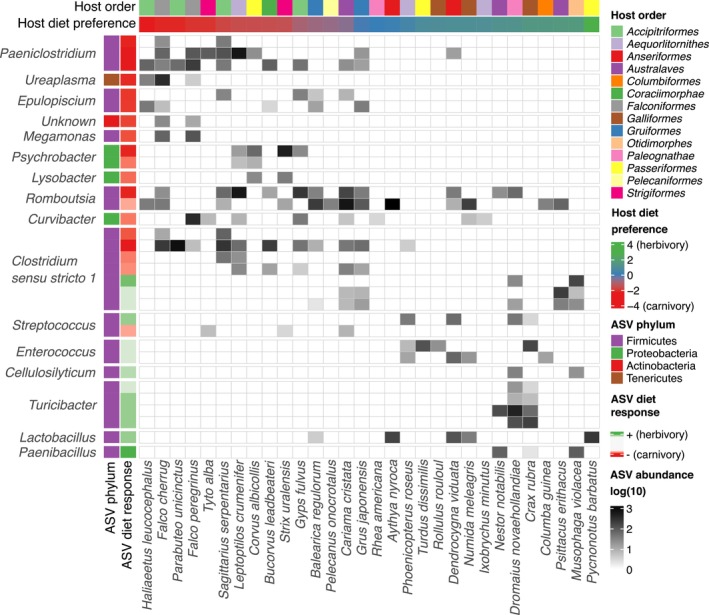
Abundance of selected amplicon sequencing variant (ASVs) grouped by genera (rows) and species (columns). Only those ASVs exhibiting high support for effect of diet preference (posterior probability > 95%) in the HMSC model are shown. Phylum classification and diet response (i.e., posterior probability of response of abundance to diet preference, with negative values representing an increase with carnivory vs. positive representing an increase with herbivory) are indicated for each ASV, and order classification and diet preference (values of first principal component) for host species.

Joint species distribution modelling estimates showed only moderate effect of host species phylogeny on presence/absence of dominant ASVs (posterior probability = 0.77), but strong support for the effect of preferred diet (posterior probability > 95%) on 32 (13 positive and 19 negative associations) of the 147 ASVs (20%) included in the model (Table S3). These comprised many common GM taxa, suggesting that diet modulates the abundance of a broad taxonomic spectrum of bacteria (Figure 4). All three ASVs from the genus *Paeniclostridium* considered in the model exhibited increased prevalence in species that prefer a carnivorous diet, while four out of five ASVs from the genus *Turicibacter* exhibited increased prevalence in host species with a vegetarian diet. In addition, we observed increased prevalence in 12 of the 16 *Bacteroidetes* ASVs in hosts depending on a vegetarian diet, but without a sufficient level of support to confirm effect of preferred diet.

**FIGURE 5:**
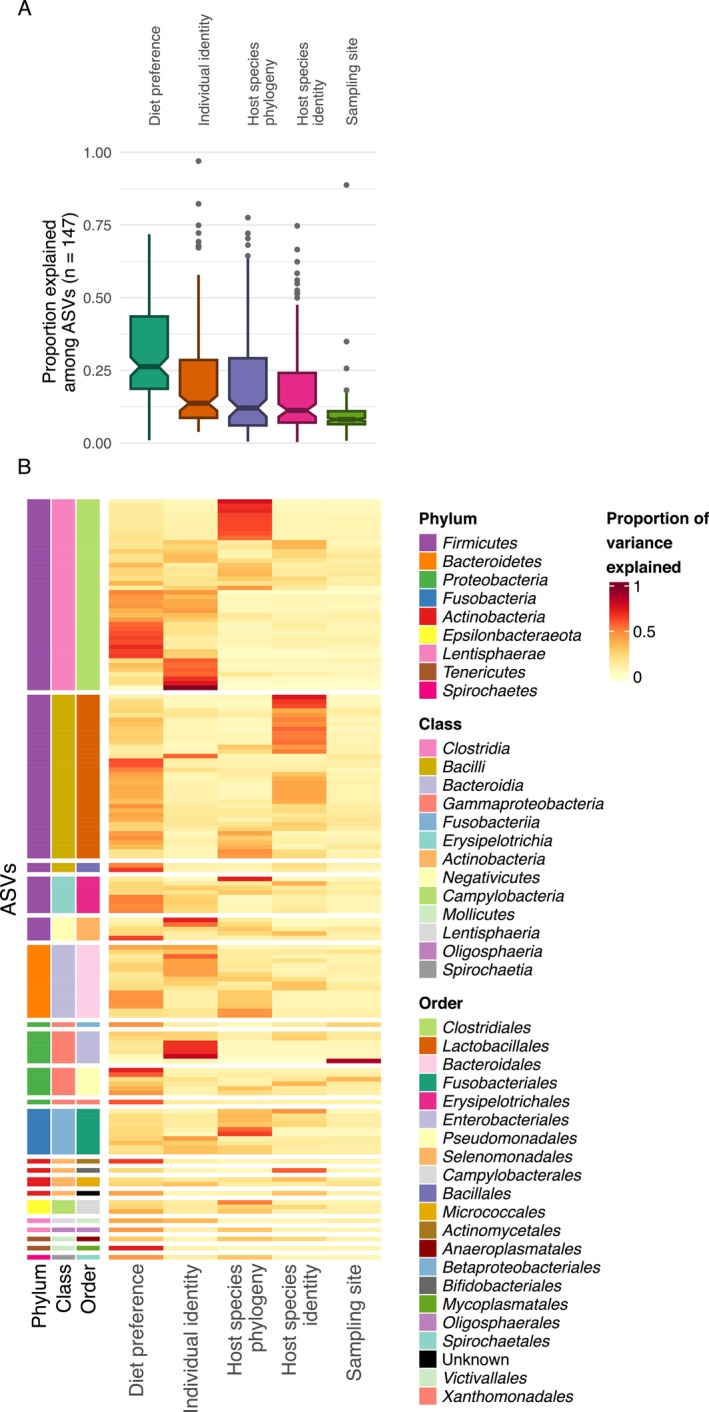
(A) Proportions of variance explained by the explanatory variables in individual amplicon sequencing variants (ASVs), summarised using boxplots. Black horizontal line represents the median, notches represent 95% confidence intervals, outliers (> 1.5 times interquartile range) represented by grey points. (B) Heatmap representation of proportion for individual ASVs, with rows split by bacterial orders.

Variance partitioning analysis revealed that diet was the most crucial factor explaining, on average, 31% of variability in ASV prevalence, followed by host species phylogeny at 19% and host species identity at 18% (Figure 5A). In contrast, sampling site explained only 9% of total variance. Results of variance partitioning analysis for individual ASVs are shown in Table S3 and Figure 5B. Finally, diet explained a high proportion of variance in ASVs from the genera *Paeniclostridium* and *Turicibacter*, while host species identity explains the greatest proportion of variability in 16 of the total 20 *Lactobacillus* ASVs.

## 4 Discussion

Research into factors that determine the composition and interspecific variability of GM in birds has focused heavily on host groups with small body size, for example, passerines. This, together with the strong dependence of GM on environmental conditions in these taxa, may bias our understanding of the drivers of variation in avian GM (Matheen et al. 2022; Sun et al. 2022; Waite and Taylor 2014), limiting the potential to generalise outcomes of such studies to the whole range of bird diversity. We focused on a set of bird species covering most clades of avian phylogeny to assess the effects of diet, locality and species identity, which, according to current literature, are the most important factors influencing avian GM (Song et al. 2020; Wang et al. 2017; Xiao et al. 2021). To achieve this, we collected and analysed a data set from host species with various dietary preferences representing a wide and balanced phylogenetic scale, including replicates of the same host species from different sampling sites (zoological gardens). Utilisation of captive individuals potentially introduces artificial effects on host GM composition, such as humanisation of GM composition, diversity decrease or homogenisation (Florkowski et al. 2023; McKenzie et al. 2017). On the other hand, zoological gardens represent more uniform habitats compared to natural conditions, reducing stochastic effects of the environment, which could mask patterns generated by other mechanisms, such as diet or phylogenetic relatedness of the host species.

Phylogenetic relatedness did not explain substantial variance in either GM alpha diversity or composition (beta diversity). On the other hand, we observed increased richness (Table 1B) and distinctive UMAP grouping of individuals in the *Paleognathae* infraclass (Figure S2D), indicating a GM variability pattern conserved among higher phylogenetic clades. However, our conclusions do not suggest global differences in GM profiles between the other two clades, that is, *Neoaves* and *Galloanserae*. Therefore, we assume that influence of phylogeny on interspecific GM variability is modest, corresponding with previous studies (Bodawatta, Koane, et al. 2021; Capunitan et al. 2020; Song et al. 2020). Our sample collection strategy allowed us to quantify and account for the stochastic environmental effect of sampling site, which was substantial for GM alpha diversity defined as ASV richness but contributed little to the variance in Shannon diversity. Sampling site also had a limited effect on the prevalence of individual ASVs. Hence, we consider environmental effects on GM variability to be weak in our setup, which could be attributed to the uniformity of environment between different zoological gardens compared to natural conditions.

The role of diet in shaping avian GM has been addressed in previous research, with a subset of studies demonstrating strong influence of diet on GM composition (Loo et al. 2019; Schmiedova et al. 2022), while others found a less significant correlation (Bodawatta, Freiberga, et al. 2021; Bodawatta, Klečková, et al. 2022). We believe that these equivocal conclusions are partially due to the heterogeneity of host taxa analysed and, in some cases, insufficient control for confounding effects. Our data, based on birds kept in zoological gardens under relatively uniform conditions, showed slight but significant changes in GM at the herbivore–carnivore scale.

These shifts were observed at the top taxonomic rank of both hosts and GM constituents. Three bacterial phyla dominated GM composition in the hosts studied (i.e., *Firmicutes*, *Proteobacteria*, *Bacteroidetes*), which is in general concordance with previous studies on wild populations (Grond et al. 2018; Sun et al. 2022). However, the proportions of these phyla exhibited pronounced variance. The most dramatic differences between host species were observed in *Bacteroidetes*, which decreased significantly in *Neoaves* compared to *Paleognathae* and *Galloanserae*, where *Bacteroidetes* were the most abundant phyla in some herbivorous species (e.g., southern screamer [ *Chauna torquata* ], ostrich [ *Struthio camelus* ]). Furthermore, beta diversity models revealed a positive association between *Bacteroidetes* and vegetarian diet. Interestingly, the phylum *Proteobacteria* exhibited the opposite trend, indicating increased abundance in carnivorous hosts and species from the *Neoaves* clade. Substitution of these two GM phyla was also confirmed by their negative covariance reported by ABDOMEN analysis. Such tendency could be explained by adaptation of bacteria in the context of different ecological adaptations of the host species groups. *Bacteroidetes* comprises many known commensal species adapted to their host's gastrointestinal tract (GIT), carrying out metabolic functions beneficial for the host, for example, complex carbohydrate digestion (Thomas et al. 2011). Therefore, host species with a complex GIT adapted to utilising a carbohydrate‐rich (vegetarian) diet, potentially profit from maintaining an association with *Bacteroidetes* (Godoy‐Vitorino et al. 2012). The non‐spore forming, mostly obligatory, anaerobic *Bacteroidetes* also rely on transgenerational transmission between hosts, which facilitates host specificity (Mazel et al. 2024). Conversely, the spore‐forming *Proteobacteria* are characterised by a diverse functional and pathogenic potential, with a still undetermined role in the bird GIT (Grond et al. 2018). These patterns, along with reduced GM richness in birds of prey and observation of host‐specific genera in vegetarian host species, are consistent with the assumptions of the ‘flight‐adapted microbiome’ hypothesis, particularly the assumption of reduced reliance on beneficial microbial functions in hosts with physiological adaptations to flight (Bodawatta, Hird, et al. 2022; Song et al. 2020). Nevertheless, direct experimental examination will be required to test these concepts, along with thorough functional determination of individual constituents of wild bird GM.

Interestingly, despite the moderate effect size, some aspects of the trajectory in observed changes resembled the more dramatic GM changes between carnivores and herbivores in mammals. As in comparative mammalian studies (Bisanz et al. 2019; Ley et al. 2008), avian GM alpha diversity and *Bacteroidetes* relative abundance tended to be reduced in carnivores. However, this compositional change was not accompanied by an increase in the abundance of *Firmicutes*, as reported for mammals. On the contrary, *Proteobacteria*, which are typically found at low abundance in mammals, tended to be more abundant in carnivorous birds. Our findings are also partially supported by study on great tits, which exhibited elevated proportion of *Proteobacteria*, but also *Bacteroidetes*, along with reduced GM richness when fed insect diet compared to seed diet (Davidson et al. 2020).

Our study also illustrated the effect of diet on bird GM at a finer taxonomic resolution, namely by the observation of striking GM convergences between several phylogenetically distant host taxa with similar food niches. This was particularly evident in birds of prey from the orders *Falconiformes* and *Accipitriformes*, which are characterised by a strictly carnivorous diet and special adaptations to prey hunting and scavenging. In addition to reduced alpha diversity, the GM profiles of these orders were characterised by the presence of specific bacterial ASVs, the prevalence of which was correlated with host diet. This pattern was most evident in the genus *Paeniclostridium* (phylum *Firmicutes*), which comprises known pathogens causing severe infections in animals and humans (Gonzalez‐Astudillo et al. 2023; Nyaoke et al. 2020). An increased prevalence of these ‘carnivore‐specific’ bacteria was also observed in other typically carnivorous hosts (marabou stork, southern ground hornbill) and, to some extent, in two crane species that rely to a greater extent on carnivory, probably explaining the similarity of these species groups in the UMAP projections. This convergence could be explained through exposure of the hosts to microbial assemblages associated with carrion (Hyde et al. 2013; Roggenbuck et al. 2014). Exceptionally, GM profiles of the Ural owl and white‐necked raven, both carnivorous species from distant orders, were found close to each other but apart from the other carnivores group. This pattern is most likely driven by the co‐occurrence of the bacterial genera *Lysobacter* and *Psychrobacter*, both of which are predominantly found in soil habitats (Christensen and Cook 1978; Kim et al. 2012), suggesting that the resemblance in GM profiles is caused by environmental contamination of the sample and not by diet preference directly. Another case of GM convergence was observed between distant herbivorous host species, namely the great curassow ( *Crax rubra* , order *Galliformes*) and the emu ( *Dromaius novaehollandiae* , order *Casuariiformes*), both sharing elevated abundance of ASVs from the genus *Turicibacter*. *Turicibacter* (phylum *Firmicutes*) is a genus of highly host‐specific bacteria, commonly found in the GM of animals, including humans, and is known for playing a role in host bile acid and lipid metabolism (Lynch et al. 2023) and production of bioactive molecules through fermentation of polysaccharides (Lin et al. 2023).

In conclusion, our results indicate a trend toward decreasing GM alpha diversity and the emergence of specific microbiota in host species with increasing preference for a carnivorous diet. This ‘carnivore‐specific’ microbiota is characterised by bacterial genera of undefined function and pathogenic potential, compared to commensal bacterial genera specific for hosts preferring a carbohydrate‐rich vegetarian diet. At this point, it is worth remarking that diet preference or trophic niche in birds is a trait correlated with phylogeny (Duque‐Correa et al. 2022). For example, there are no hypercarnivorous representatives among the *Paleognathae*, or any strict herbivore among birds of prey. This phenomenon prevents direct testing whether phylogeny or diet is the main driver of interspecific variability in bird GM. While diet and host phylogeny showed only a moderate correlation in our data (Pagel's *λ* = 0.6 and 0.4, respectively), and the statistical methods we used were specifically designed to separate these effects, we cannot completely rule out that our models may struggle to quantify the variance explained by these two factors. To enable differentiation of these effects, experimental studies will be needed that control for host phylogeny and manipulate diet, as in Bodawatta, Freiberga, et al. (2021); however, such studies will be difficult or impossible to undertake in natural environments. Alternatively, evolutionary conserved diet can be considered as a standalone factor combining effects of host phylogeny and diet preference. Consequently, further analyses on taxonomically broader datasets derived from a range of habitats will be required to account for the exceptional diversity of birds in terms of life‐history traits, evolutionary background and ecological characteristics.

## Author Contributions

**Jan Kubovčiak:** conceptualization (equal), formal analysis (lead), funding acquisition (equal), investigation (lead), methodology (lead), project administration (lead), writing – original draft (lead), writing – review and editing (equal). **Jakub Kreisinger:** conceptualization (equal), data curation (lead), formal analysis (supporting), funding acquisition (equal), methodology (supporting), writing – review and editing (equal).

## Conflicts of Interest

The authors declare no conflicts of interest.

## Supporting information


**Figure S1:** Distribution of diet preference and alpha diversity indices between sampling sites. Black horizontal line represents the median, outliers (> 1.5 times interquartile range) represented by grey points.

**Figure S2:** Uniform manifold approximation and projection (UMAP) projection of GM profiles similarities with samples coloured by (A) sampling site, (B) date of DNA isolation, (C) daytime hour, (D) host clade assignment.

**Figure S3:** ABDOMEN prediction of ancestral GM composition for top five most abundant phyla.

**Figure S4:** Covariances between bacterial phyla estimated by ABDOMEN (mean of the posterior distribution). Star indicates significant value (0 not included in 95% CI).

**Table S1:** (A) Sample metadata, with alpha diversity indices and diet preference values. (B) Diet rations provided to host species in individual zoological gardens.

**Table S2:** Results of all Phylogenetic Generalised Linear Mixed Models explaining variation in (A) alpha diversity indices, (B) abundance of three most represented phyla, and (C) alpha diversity indices among sampling sites. Significant *p* values (< 0.05) are highlighted with a yellow background.

**Table S3:** Proportions of variance in prevalence of tested amplicon sequencing variants explained by individual effects in joint species distribution models and posterior probabilities for positive or negative response to diet preference.

## Data Availability

Raw sequence reads are deposited in the European Nucleotide Archive (project accession number: PRJEB83978).
