# Supplementary figures and images for: Host Diet Preference Drives Diversity and Composition of Gut Microbiota in Captive Birds
Source: Ecol Evol. 2025 Nov 11;15(11):e72463. doi: 10.1002/ece3.72463 (PMC12611309; doi:10.1002/ece3.72463)

Figure S1

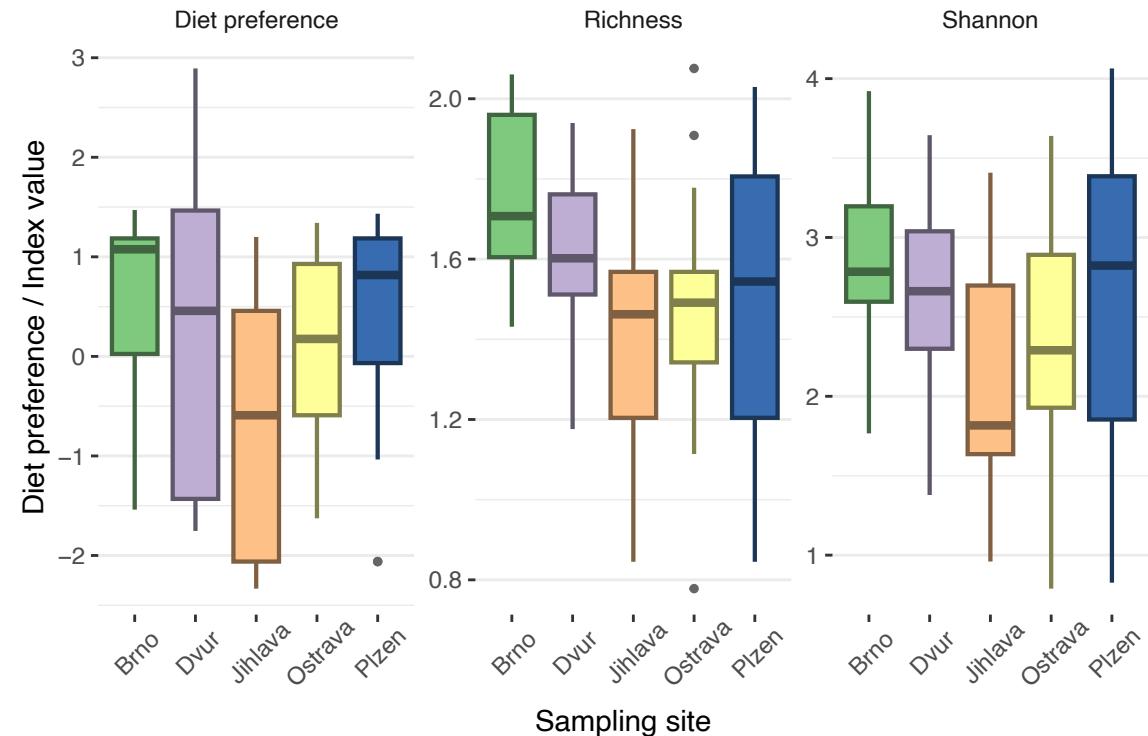

Supplement: Supplementary file 1 — Figure S1: Distribution of diet preference and alpha diversity indices between sampling sites. Black horizontal line represents the median, outliers (> 1.5 times interquartile range) represented by grey points. [file ECE3-15-e72463-s003.pdf]

Figure S2

A

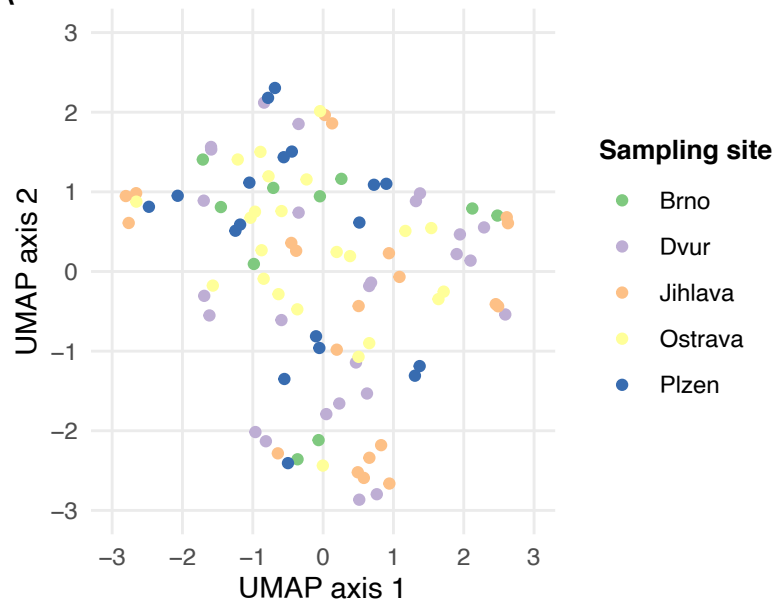

B

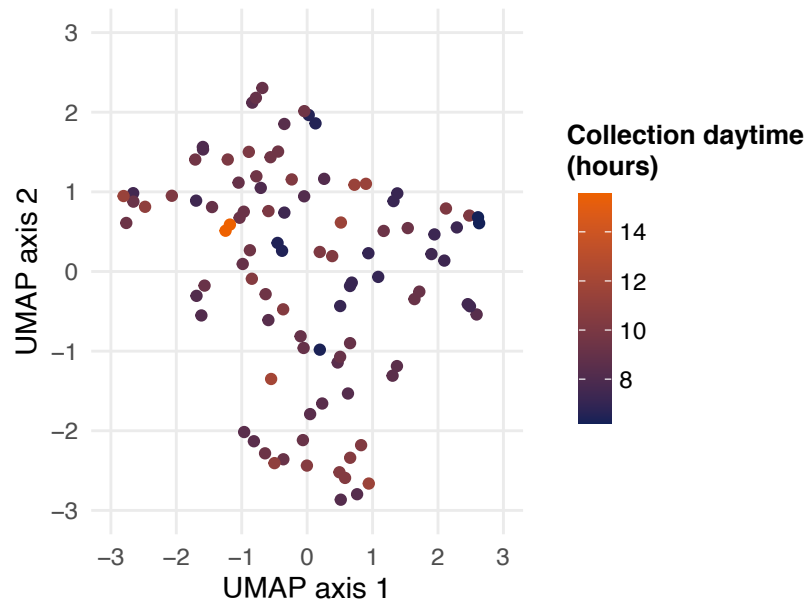

C

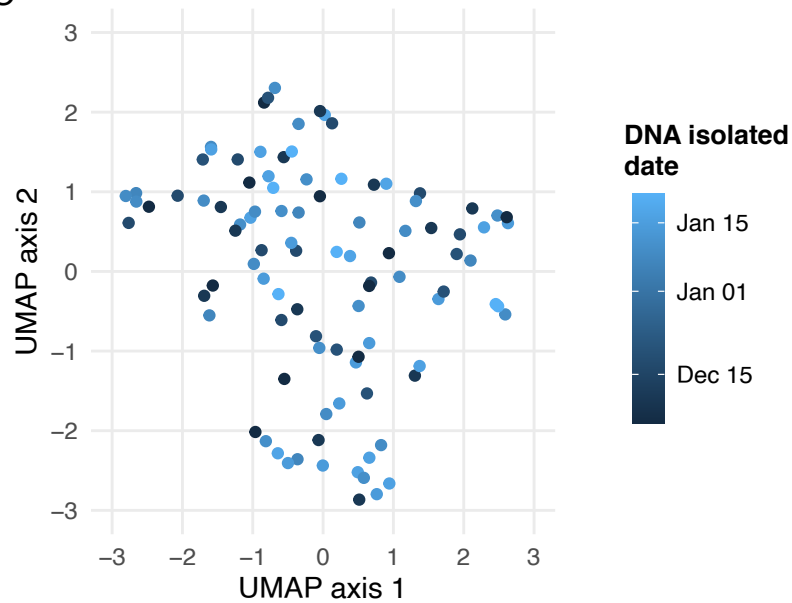

D

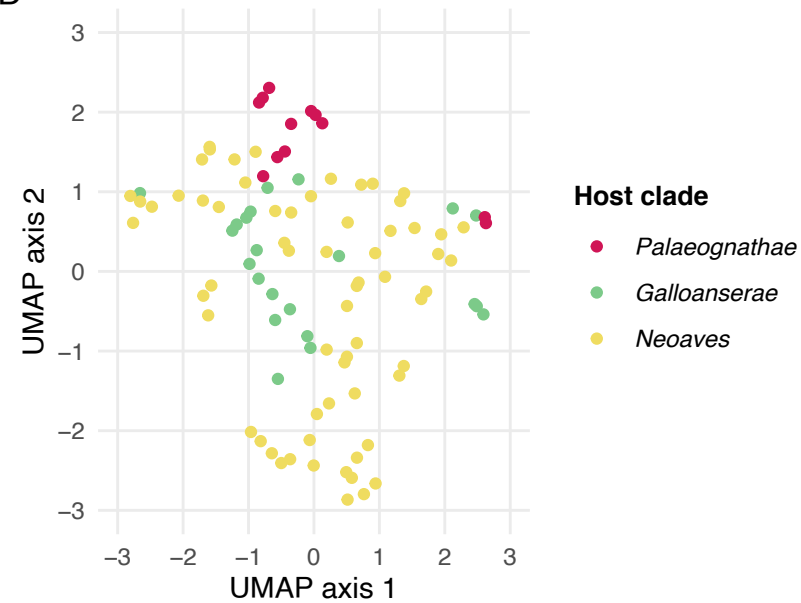

Supplement: Supplementary file 2 — Figure S2: Uniform manifold approximation and projection (UMAP) projection of GM profiles similarities with samples coloured by (A) sampling site, (B) date of DNA isolation, (C) daytime hour, (D) host clade assignment. [file ECE3-15-e72463-s007.pdf]

Figure S3

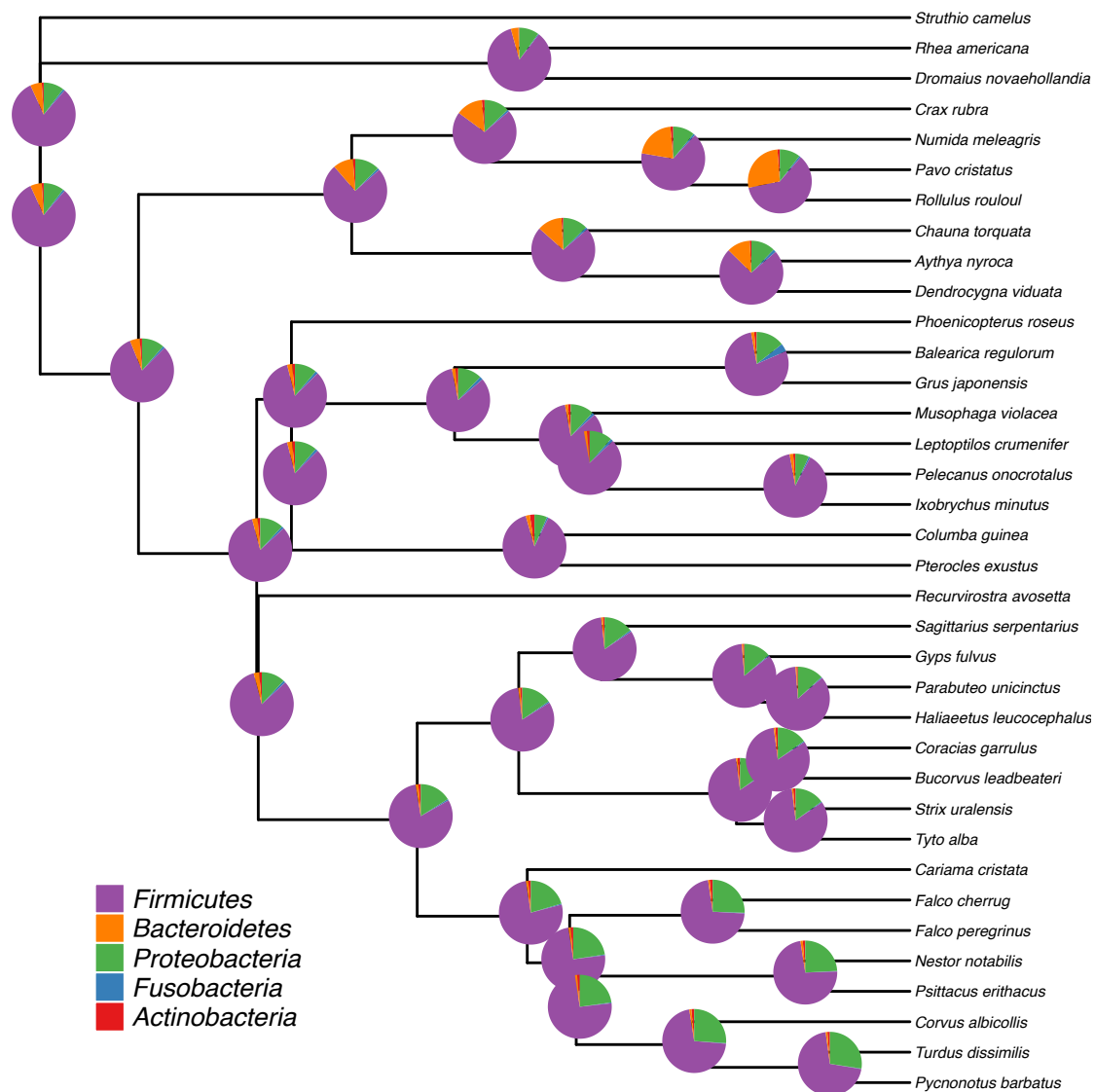

Supplement: Supplementary file 3 — Figure S3: ABDOMEN prediction of ancestral GM composition for top five most abundant phyla. [file ECE3-15-e72463-s004.pdf]

Figure S4

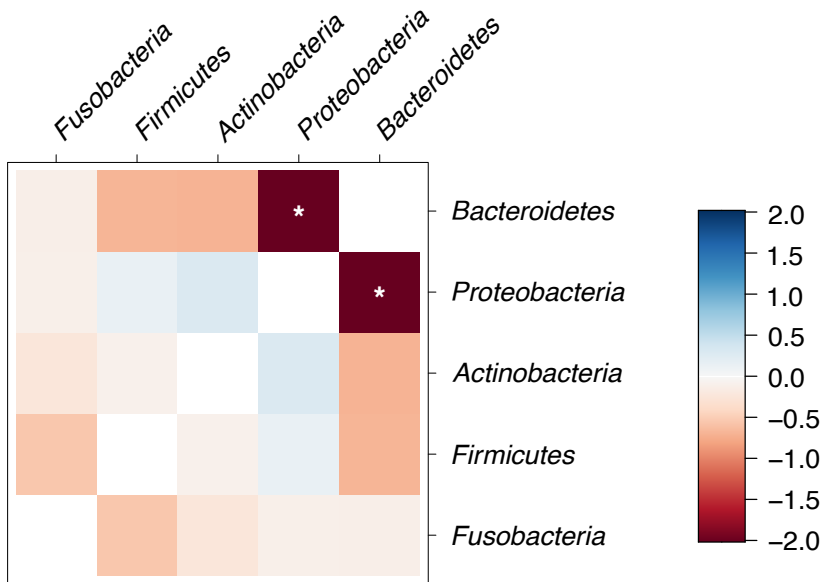

Supplement: Supplementary file 4 — Figure S4: Covariances between bacterial phyla estimated by ABDOMEN (mean of the posterior distribution). Star indicates significant value (0 not included in 95% CI). [file ECE3-15-e72463-s006.pdf]
